# CT-Guided Liver Biopsy: Evaluation of Spectral Data From Dual-Layer Detector CT for Improved Lesion Detection

**DOI:** 10.1007/s00270-023-03550-7

**Published:** 2023-09-27

**Authors:** Andreas P. Sauter, Roland Proksa, Andreas Knipfer, Stefan Reischl, Rickmer F. Braren, Jonathan Nadjiri, Felix Kopp, Peter B. Noël, Markus R. Makowski, Ernst J. Rummeny, Alexander A. Fingerle

**Affiliations:** 1 Department of Diagnostic and Interventional Radiology, School of Medicine and Klinikum Rechts Der Isar, Technical University of Munich, Ismaningerstr. 22, 81675 Munich, Germany; 2 Philips Research, Hamburg, Germany; 3 Department of Radiology, Helios Klinikum München West, Munich, Germany; 4 Department of Interventional Radiology, School of Medicine and Klinikum Rechts Der Isar, Technical University of Munich, Munich, Germany; 5 Department of Radiology, Perelman School of Medicine, University of Pennsylvania, Philadelphia, USA; 6 Department of Radiology, Kantonsspital Münsterlingen, Muensterlingen, Switzerland

**Keywords:** Computed tomography, X-Ray computed tomography, Image-guided biopsy, Dual-layer spectral CT, Liver intervention, Image post-processing technique

## Abstract

**Purpose:**

Evaluation of dual-layer spectral computed tomography (CT) for contrast enhancement during image-guided biopsy of liver lesions using virtual monoenergetic images (VMI) and virtual non-contrast (VNC) images.

**Methods:**

Spectral CT data of 20 patients receiving CT-guided needle biopsy of focal liver lesions were used to generate VMI at energy levels from 40 to 200 keV and VNC images. Images were analyzed objectively regarding contrast-to-noise ratio between lesion center (CNR_cent_) or periphery (CNR_peri_) and normal liver parenchyma. Lesion visibility and image quality were evaluated on a 4-point Likert scale by two radiologists.

**Results:**

Using VMI/VNC images, readers reported an increased visibility of the lesion compared to the conventional CT images in 18/20 cases. In 75% of cases, the highest visibility was derived by VMI-40. Showing all reconstructions simultaneously, VMI-40 offered the highest visibility in 75% of cases, followed by VNC in 12.5% of cases. Either CNR_cent_ (17/20) or/and CNR_peri_ (17/20) was higher (CNR increase > 50%) in 19/20 cases for VMI-40 or VNC images compared to conventional CT images. VMI-40 showed the highest CNR_cent_ in 14 cases and the highest CNR_peri_ in 12 cases. High image quality was present for all reconstructions with a minimum median of 3.5 for VMI-40 and VMI-50.

**Conclusions:**

When implemented in the CT scanner software, automated contrast enhancement of liver lesions during image-guided biopsy may facilitate the procedure.

**Graphical Abstract:**

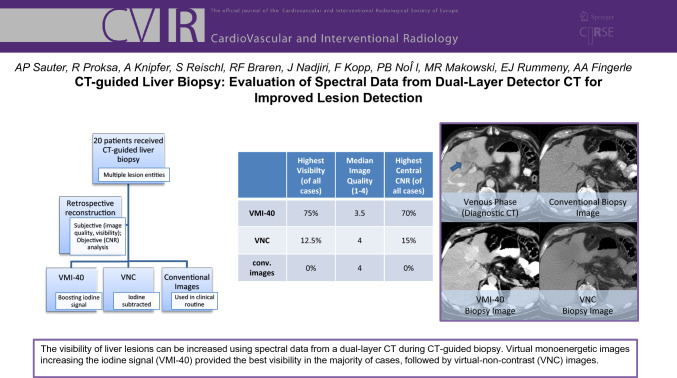

## Introduction

In clinical practice, focal liver lesions are a common finding. In most cases, diagnostic imaging enables the differentiation between benign and malignant focal liver lesions [1, 2]. However, if lesions show atypical features or growth, biopsy may be warranted to perform histological analysis [3]. Biopsies are commonly performed using either ultrasound guidance or computed tomography (CT) imaging. Although ultrasound is the preferred imaging modality for liver biopsy, there are limitations [4]. In comparison, CT images provide a better overview, and the biopsy needle is clearly visible due to its high density [5]. Intravenous contrast is often needed for the detection of liver lesions. However, the visualization of focal liver lesions provided by intravenous contrast can be short-lived due to an increasing equilibrium of the lesion and the liver parenchyma [5]. In these cases, it would be helpful to prolong and enhance the contrast between the lesion and the normal liver tissue to facilitate the biopsy procedure. In recent years, dual-energy CT (DE-CT) has demonstrated that the derived spectral information can improve the detectability of focal liver lesions. DE-CT uses the absorption characteristics for the differentiation and quantification of materials while conventional CT only quantifies the attenuation of the total X-ray spectrum reaching the detector [6]. In dual-layer detector CT (DL-CT), the spectral separation occurs at the detector level offering several advantages compared to source-based approaches [7]. For DL-CT, spectral data are acquired additionally to the conventional images with every scan and retrospective reconstruction of spectral information is possible [8]. Using these spectral data, iodine can be quantified accurately [9]. Virtual non-contrast (VNC) images can be created via detection and quantification of iodine, which is then subtracted, resulting in a calculated unenhanced image without iodine [10, 11]. Furthermore, virtual monoenergetic images (VMIs) can be calculated, simulating monoenergetic X-ray source during the acquisition of the CT scan [7], boosting the iodine signal at low voltages and decreasing it at high voltages [12].

The objective of our retrospective study was to evaluate the potential benefit of VMI and VNC to prolong and improve lesion detectability during liver biopsy following a contrast-enhanced planning CT scan.

## Materials and Methods

### Patient Population

Institutional review board approval was obtained for this retrospective study. Informed consent was waived by the institutional review board as no additional data besides clinical obtained images were used. All examinations were performed exclusively for clinical use. Patients were referred for biopsy by other departments of the hospital.

Twenty consecutive patients who were examined with a DL-CT and received a triphasic CT before biopsy were retrospectively included in the current study. Median patient age was 63.9 (36.1–84.7) years, with 13 males and 7 females. A core needle biopsy of a hepatic lesion was performed in all patients. The images obtained during the biopsies were analyzed retrospectively. Biopsies were performed with conventional CT images. Biopsy outcomes are shown in Table 1.

**Table 1:** Patient characteristics and biopsy outcomes

pat #	Dob	Sex	Histology
1	24.02.1933	f	Intestinal-type adenocarcinoma
2	05.03.1940	f	Neuroendocrine tumor (ileum)
3	18.06.1976	m	Small-cell neuroendocrine carcinoma
4	18.10.1945	m	Ductal adenocarcinoma (metastasis of pancreas carcinoma)
5	30.05.1946	f	Adenocarcinoma of pancreatobiliary origin
6	29.02.1944	m	Adenocarcinoma of pancreatobiliary origin
7	23.12.1981	f	Metastasis of breast cancer
8	01.01.1963	m	Neuroendocrine carcinoma
9	06.02.1975	f	Metastasis of uveal melanoma
10	11.03.1947	m	Fibrosis with necrosis
11	08.10.1957	f	Metastasis of breast cancer
12	25.01.1945	m	Metastasis of prostate cancer
13	17.09.1943	m	Carcinoma, not further classifiable
14	15.10.1951	m	Ductal adenocarcinoma (metastasis of pancreas carcinoma)
15	18.06.1976	m	Adenoid squamous cell carcinoma
16	10.03.1956	m	Metastasis of prostate cancer
17	21.05.1950	m	Fibrosis
18	27.02.1959	m	Adenocarcinoma of pancreatobiliary origin
19	03.10.1949	f	Adenocarcinoma of the ovary
20	26.11.1942	m	Adenocarcinoma of pancreatobiliary origin

### Image Acquisition

All examinations were performed using a DL-CT (IQon; Philips Healthcare). Patients underwent a triphasic CT scan of the abdomen that included a non-enhanced scan, an arterial phase and a venous phase (75 s after contrast injection) at 120 kVp and 64 × 0.625 mm detector configuration. An adjustment of the tube current based on the scout view (z-axis modulation) is included by default. Iodinated contrast media was injected for the arterial phase (1.2 ml/kg with a maximum of 120 ml) followed by a 50-ml saline chaser. After the triphasic CT scan, the core needle biopsy was planned based on the acquired data. The scanner couch was centered in the chosen position, the image acquisition during the biopsy included three slices (head-mid-foot) with a slice thickness of 3 mm. Following the biopsy, a delayed phase without intravenous contrast was performed to rule out hemorrhage. If a substantial hemorrhage was seen, additional scans with intravenous contrast would have been added, however, this was not the case in the study population. All data sets were reconstructed in axial view with slice thickness of 3 mm and a 512 × 512 image matrix.

For each phase as well as for the images during biopsy, spectral data were acquired. Using these data, virtual monoenergetic images (VMIs) at tube currents of 40–200 keV with steps of 10 keV (VMI40–VMI200) as well as virtual non-contrast images (VNC) were calculated using the commercially available spectral workstation (IntelliSpace Portal (v. 8.0.2), Philips Healthcare). Additionally, conventional images (CIs), which are used in clinical routine, were saved. Hereby, CIs are the images of the respective phase, which are acquired without using image modification via spectral information.

### Objective Image Analysis

For each patient, one hepatic lesion was chosen for biopsy. This lesion was analyzed in all phases (unenhanced, arterial, venous and delayed) as well as in the biopsy images. Regions of interests (ROIs) were defined in the center of the lesion, in the periphery of the lesion as well as in normal hepatic parenchyma. Hereby, the ROIs were drawn as large as possible. For every ROI, the mean Hounsfield units (HU) and the corresponding standard deviation (SD) were measured. For the center and the periphery of each measured lesion, the contrast-to-noise ratio (CNR) were calculated using the formula:

CNR = (HU_lesion_-HU_liver_)/SD_liver_.

where HU_lesion_ is the HU-value of the lesion (center or periphery), HU_liver_ is the HU-value of the normal liver parenchyma and SD_liver_ is the standard deviation in the normal liver parenchyma, representing noise.

### Reader Study

To evaluate the subjective image quality as well as the visibility of the focal lesion, two radiologists with 3 and 7 years of experience in CT-guided interventions were asked to analyze the biopsy images in all reconstructions (VMI40-VMI200, VNC, CI). Readers were allowed to change window leveling at their preference to prevent an influence of a predefined window level.

First, each reconstruction was shown separately. Here, the subjective image quality (1-very low; 2-poor; 3-good; 4-very good) and the visibility of the focal lesion (1-not visible; 2-poor; 3-good; 4-very good) were rated on a 4-point Likert scale.

Afterward, all reconstructions were shown simultaneously, and the readers were asked to rate the top 3 reconstructions enabling the best visibility of the focal lesion. Here, the total number of ratings is shown, so a maximum of 120 ratings (20 cases, 2 readers, 3 ratings per reader and case) are possible. Thus, 40 ratings were given for top-1, top-2 and top-3, respectively.

### Statistical Analysis

Statistical analysis was performed by dedicated software packages (SPSS, IBM; Excel 2016, Microsoft). CNR is given in mean ± (SD), these data were tested for Gaussian distributions via D'Agostino-Pearson omnibus test. As Gaussian distribution was present, differences were tested using a paired t-test. For the reader study, the median of both readers was calculated for each subset. Then, differences between the different reconstructions were tested using the Wilcoxon signed-rank test. For each test, *p* value < 0.05 indicated statistical significance.

## Results

### Reader Study

Analyzing individual biopsy images (showing one reconstruction at a time), the highest visibility was found for VMI-40 in 75% of cases (Table 2). This results in a median visibility of 3 for VMI-40 over all cases. Hereby, the difference between VMI-40 and all other reconstructions (apart from VMI-50) as well as between VMI-50 and all other reconstructions (apart from VMI-40 and VMI-60) was significant (*p* < 0.05).

**Table 2:** Results of the reader study

pat #	CI	VMI 40	VMI 50	VMI 60	VMI 70	VMI 80	VMI 90	VMI 100	VMI 110	VMI 120
1	1.5	1.5	1.5	1.5	1.5	1.5	1.5	1.5	1.5	2
2	1	**1.5**	**1.5**	1	1	1	1	1	1	1
3	1	**3**	2	2	1.5	1	1	1	1	1
4	3	**4**	**4**	3.5	3	3	3	2.5	2.5	2.5
5	1	**3.5**	2.5	2	2	1.5	1	1	1	1
6	1.5	1	**2**	2	2	2	2	2	1.5	2
7	1.5	1	1	1	1.5	2	2	2	2	2
8	3	**3.5**	**3.5**	**3.5**	**3.5**	3	2.5	2.5	2.5	2.5
9	2	**2.5**	2	2	2	2	2	2	2	2
10	2.5	**4**	**4**	3.5	3	2.5	2.5	2.5	2	2
11	3	2	2	3	3	3	3	3	3	3
12	2.5	**3.5**	3	3	3	2.5	2.5	2.5	2.5	2.5
13	2	**3**	**3**	**3**	2.5	2	2	2	2	2
14	1.5	**3**	2.5	2	2	1	1	1	1	1
15	1	**3**	2	2	1.5	1	1	1	1	1
16	1	**2.5**	**2.5**	2	2	1.5	1	1	1	1
17	1	**1.5**	**1.5**	1	1	1	1	1	1	1
18	1.5	**3**	3	2.5	2.5	1.5	1.5	1.5	1.5	1.5
19	1	1	1	1	1	1	1	1	1	1
20	1.5	**4**	3.5	3	2	2	1.5	2	2	2
median	1.5	3	2.5	2	2	1.75	1.5	1.75	1.5	2

pat #	VMI 130	VMI 140	VMI 150	VMI 160	VMI 170	VMI 180	VMI 190	VMI 200	VNC
1	2	2	2	2	2	2	2	2	2
2	1	1	1	1	1	1	1	1	1
3	1.5	1.5	1.5	1.5	2	2	2	2	2
4	2.5	2.5	2.5	2	2	2	2	2	2
5	1	1	1	1	1	1	1	1	1
6	2	2	2	2	2	2	2	2	2
7	**2.5**	**2.5**	**2.5**	**2.5**	**2.5**	**2.5**	**2.5**	**2.5**	**2.5**
8	2	2	2	2	2	2	2	2	2
9	2	2	2	2	2	2	2	2	2
10	2	2	1.5	1.5	1.5	1.5	1.5	1.5	1.5
11	3	3	3	3	3	3	3	2.5	2.5
12	2.5	2.5	2.5	2.5	2.5	2.5	2.5	2.5	2.5
13	2	2	2	2	2	2	2	2	2
14	1	1	1	1	1	1	1	1	1
15	1.5	1.5	1.5	1.5	2	2	2	2	2
16	1	1	1	1	1	1	1	1	1
17	1	1	1	1	1	1	1	1	1
18	1.5	1.5	1.5	1.5	1.5	1.5	1.5	1.5	1.5
19	1	1	1	1	1	1	1	1	1
20	2	2	2	2	2	2	2	2	2
median	2	2	1.75	1.75	2	2	2	2	2

Results for the visibility of the focal liver lesions in the biopsy images for conventional images (CIs), virtual monoenergetic (VMI) images at 40–200 keV and virtual non-contrast images (VNC) are shown. Results for each patient (1–20) are presented individually as well as the median of all patients. Highest ratings regarding visibility of the lesions are highlighted in bold. For patient #11 and #19, no rating is highlighted as almost all ratings were identical

Median image quality was 3.5 for VMI-40 and VMI-50. For all other reconstructions, median image quality was 4. This results in a significant difference (*p* < 0.05) of VMI-40 as well as VMI-50 compared to all other reconstructions.

Rating the best three reconstructions regarding visibility when all reconstructions were shown simultaneously, VMI-40 was rated as top-one 30 times, VMI-50 two times and VNC five times. VMI-70 and VMI-140 were rated top-one once, respectively. One case (patient 19) could not be rated by one reader as all reconstructions appeared similar to him.

**Fig. 1:**
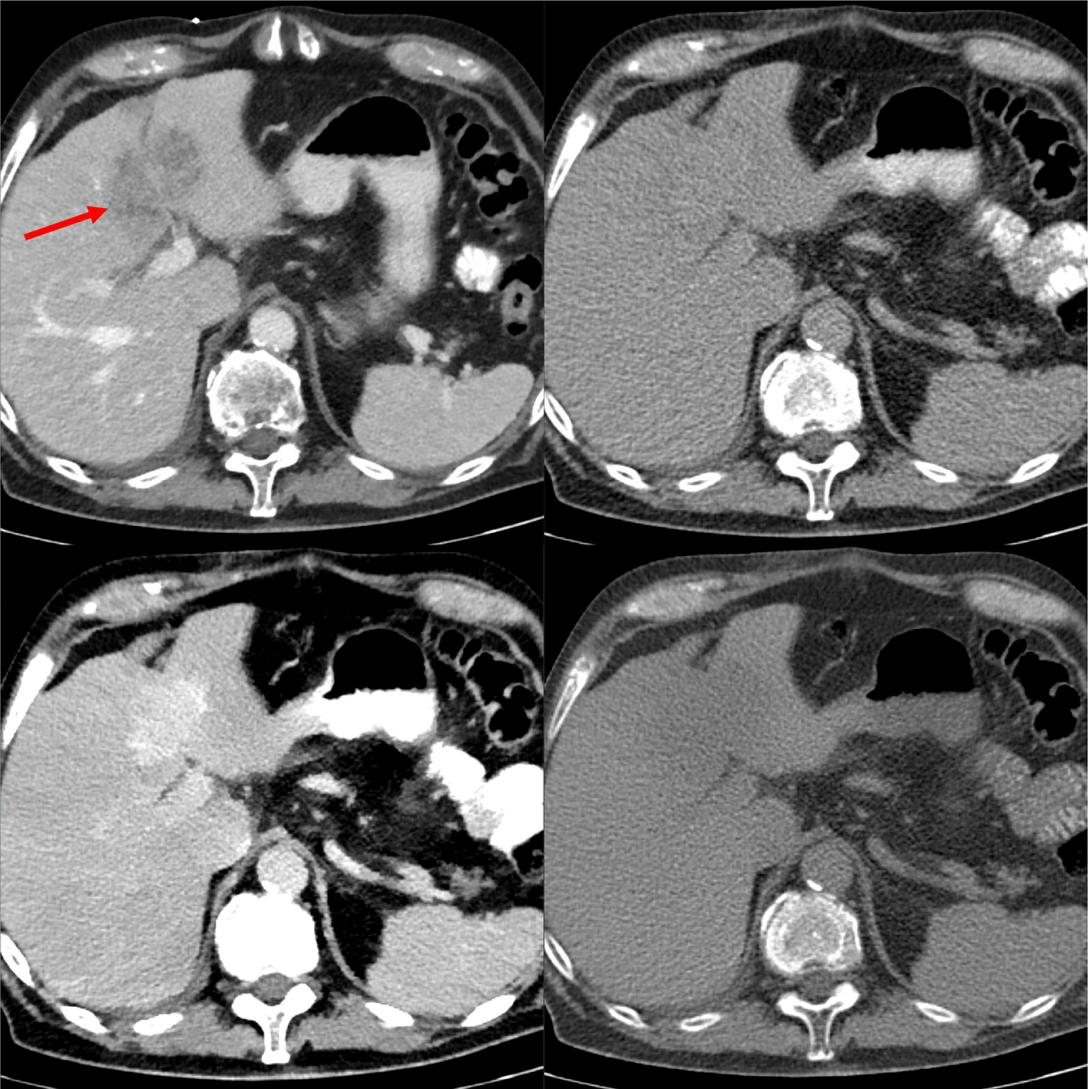
Patient #20 with a primary intrahepatic cholangiocarcinoma. Shown are the diagnostic CT in the venous phase (top left), as well as the corresponding biopsy images. The lesion is best visible in VMI-40 image (bottom left) due to hyperdensity but also well visible in the VNC image (bottom right) due to subtraction of iodine (hypodensity). The conventional reconstruction (top right) shows the worst visibility

**Fig. 2:**
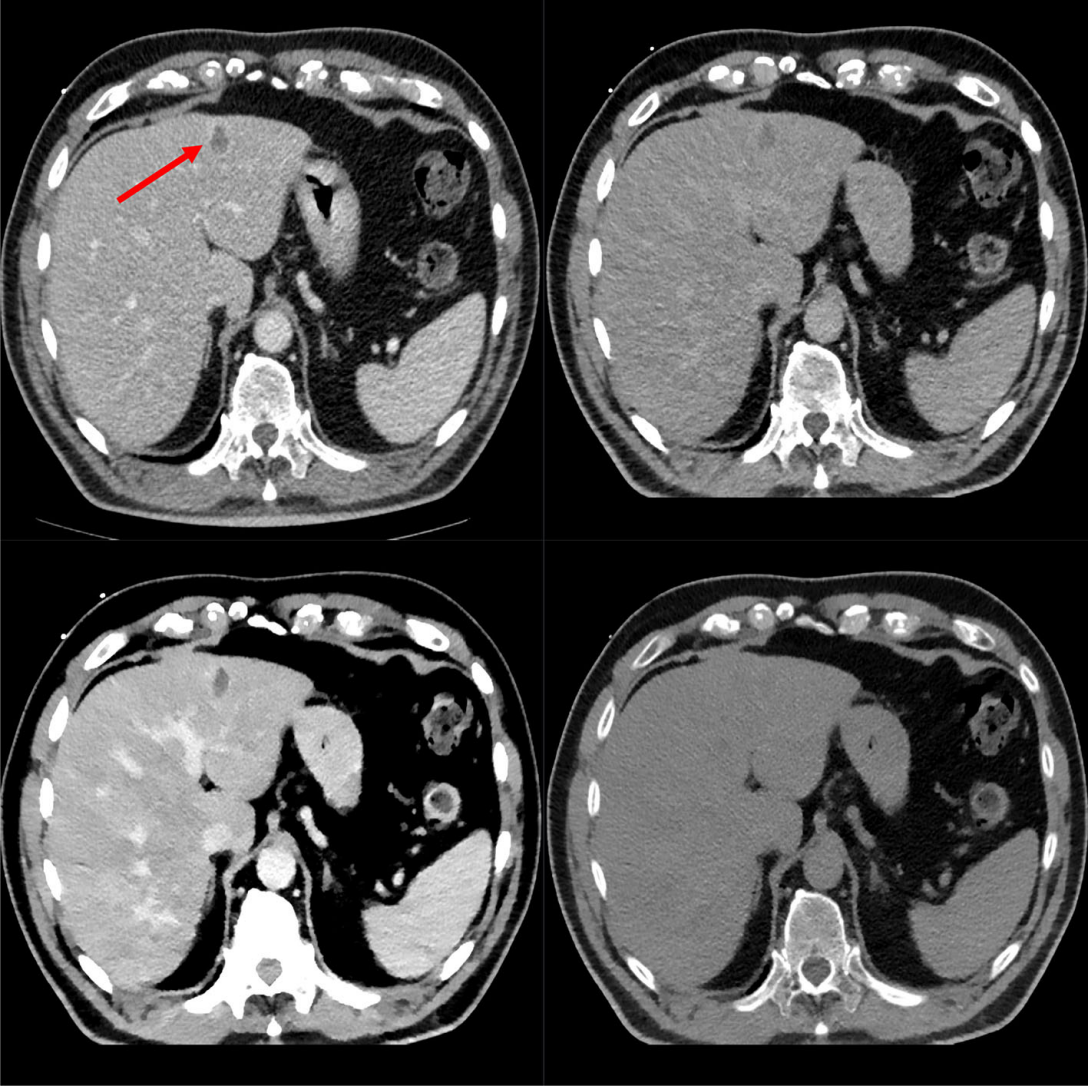
Patient #10, the shown lesion was histopathologically identified as necrosis. Shown are the diagnostic CT in the venous phase (top left), as well as the corresponding biopsy images. The lesion is best visible in the VMI-40 image (bottom left) due to the missing iodine uptake and thus hypodensity compared to the remaining liver parenchyma. It is also visibility in the conventional reconstruction (top right) and worst visible in the VNC image (bottom right)

**Fig. 3:**
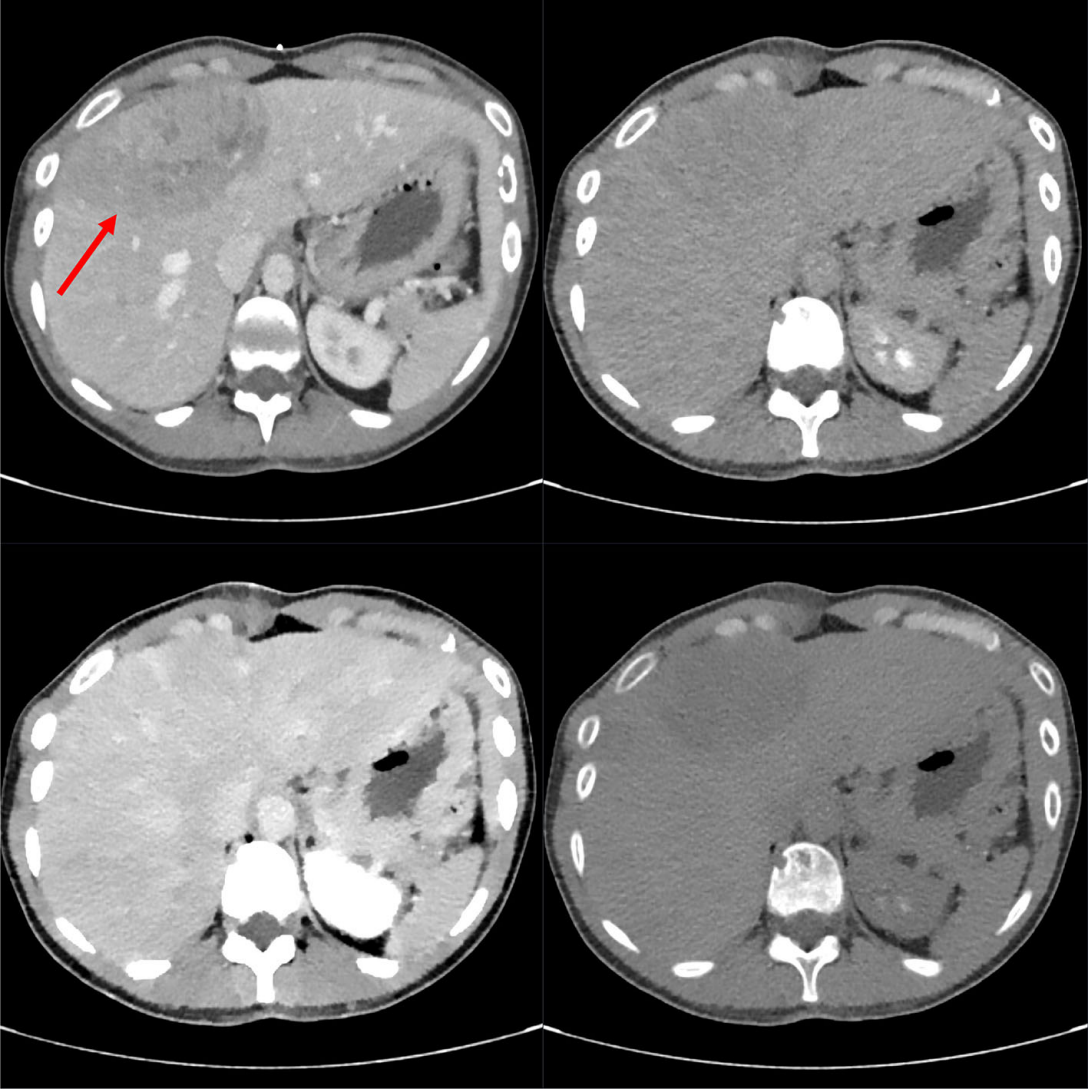
Patient #7, the shown lesion was histopathologically identified as a breast cancer metastasis. Shown are the diagnostic CT in the venous phase (top left), as well as the corresponding biopsy images. The lesion is best visible in the VNC image (bottom right) due to hypodensity compared to the remaining liver parenchyma. It is also visibility in the conventional reconstruction (top right) and worst visible in the VMI-40 image (bottom left)

The biopsy images of three patients are shown in Figs. 1, 2, 3. Figure 1 shows the example of a lesion with the best visibility in VMI-40 images due to hyperdensity. Figure 2 shows a lesion with best visibility in VMI-40 images, and Fig. 3 shows a lesion with best visibility in VNC images. In all examples, the lesion is clearly better visible in spectral images than in conventional images.

### Objective Image Analysis

The healthy liver parenchyma was then compared to the center (Table 3) and to the periphery (Table 4) of the selected lesion. The comparison of the CNR values of the lesion and the healthy parenchyma showed ratios of 1–62 (periphery of the lesion) and 1–136 (center of the lesion). Over all cases, VMI-40 showed the highest mean contrast for center (3.40) and periphery (2.53), whereas the contrast in CI was 1.68 and 1.03, respectively. The difference between VMI-40 and any other reconstruction was significant for the center and the periphery of the lesion (*p* < 0.05). For the center of the lesion, VMI-40 showed the highest ratio in 14 cases and for the periphery in 12 cases, respectively. VMI-40 showed the highest ratio in either the center or the periphery in all but one case. VNC showed the highest ratio for the center in three cases and for the periphery in four cases, respectively.

**Table 3:** Results of the objective image analysis comparing the contrast-to-noise ratio of the lesions’ center and normal liver parenchyma in the biopsy image

CNR: center of lesion vs. liver	CI	VMI 40	VMI 50	VMI 60	VMI 70	VMI 80	VMI 90	VMI 100	VMI 110	VMI 120
pat #										
1	0.04	**1.12**	0.54	0.16	0.08	0.25	0.35	0.42	0.48	0.51
2	0.02	**2.72**	1.63	0.79	0.34	0.01	0.18	0.35	0.43	0.49
3	2.78	3.28	3.56	3.69	3.79	3.86	3.85	3.85	3.85	3.85
4	3.39	**6.00**	5.11	4.55	4.19	3.94	3.79	3.71	3.62	3.55
5	3.57	**11.40**	8.89	6.73	5.29	4.30	3.56	3.10	2.74	2.46
6	3.06	**4.46**	4.05	3.88	3.71	3.56	3.51	3.44	3.41	3.41
7	0.86	1.54	0.31	0.45	0.88	1.20	1.39	1.49	1.59	1.69
8	4.41	**7.32**	6.17	5.38	4.95	4.71	4.47	4.36	4.17	4.19
9	0.84	**2.33**	1.83	1.50	1.27	1.14	1.03	0.97	0.92	0.88
10	1.55	**4.96**	3.54	2.54	1.91	1.49	1.22	1.05	0.91	0.81
11	0.92	0.37	0.94	1.35	1.59	1.74	1.83	1.91	1.97	2.00
12	5.91	**8.93**	8.10	7.46	7.09	6.80	6.72	6.49	6.48	6.37
13	2.71	2.72	2.83	2.85	2.86	2.87	2.88	2.88	2.86	2.88
14	0.28	**1.78**	0.98	0.43	0.10	0.12	0.27	0.37	0.44	0.49
15	0.42	**2.43**	1.10	0.27	0.26	0.60	0.81	0.96	1.06	1.14
16	0.72	0.78	0.80	0.81	0.81	0.81	0.79	0.80	0.80	0.80
17	0.42	**2.46**	1.56	0.98	0.61	0.37	0.22	0.11	0.03	0.02
18	1.42	1.02	0.11	0.86	1.32	1.61	1.79	1.93	2.03	2.10
19	0.04	**0.50**	0.35	0.26	0.20	0.16	0.14	0.12	0.11	0.10
20	0.16	**1.91**	0.89	0.26	0.13	0.39	0.53	0.66	0.73	0.78

CNR: center of lesion vs. liver	VMI 130	VMI 140	VMI 150	VMI 160	VMI 170	VMI 180	VMI 190	VMI 200	VNC	CNRmax/CNRconv
pat #										
1	0.53	0.56	0.58	0.58	0.6	0.6	0.62	0.62	0.68	28
2	0.56	0.64	0.71	0.54	0.71	0.65	0.71	0.71	0.90	136
3	3.91	3.87	3.94	3.85	3.90	3.94	3.92	3.87	3.94	1.4
4	3.52	3.51	3.45	3.46	3.42	3.41	3.44	3.44	3.28	1.8
5	2.30	2.16	2.04	1.97	1.88	1.83	1.72	1.70	1.74	3.2
6	3.40	3.38	3.33	3.38	3.31	3.30	3.36	3.35	3.25	1.5
7	1.66	1.70	1.81	1.76	1.85	1.78	1.90	1.83	**1.96**	2.3
8	4.11	4.10	4.07	3.98	4.02	3.94	3.96	3.98	3.84	1.7
9	0.87	0.84	0.82	0.82	0.82	0.78	0.80	0.80	0.72	2.8
10	0.74	0.70	0.65	0.63	0.58	0.55	0.55	0.55	0.44	3.2
11	2.04	2.02	2.05	2.06	2.06	2.11	2.06	2.09	**2.16**	2.3
12	6.36	6.24	6.38	6.20	6.29	6.31	6.21	6.22	6.17	1.5
13	2.87	2.82	2.89	2.82	2.87	2.87	2.81	2.87	2.88	1.1
14	0.52	0.56	0.58	0.59	0.61	0.62	0.63	0.63	0.72	6.4
15	1.19	1.24	1.28	1.30	1.32	1.34	1.35	1.36	1.49	5.8
16	0.80	0.78	0.80	0.78	0.81	0.79	0.77	0.79	0.81	1.1
17	0.07	0.10	0.12	0.13	0.16	0.17	0.18	0.20	0.29	5.9
18	2.15	2.20	2.21	2.26	2.27	2.27	2.27	2.33	**2.43**	1.7
19	0.10	0.09	0.08	0.08	0.08	0.08	0.07	0.07	0.06	12.5
20	0.82	0.85	0.89	0.90	0.93	0.92	0.94	0.96	1.04	11.9

CNR for conventional images (CIs), virtual monoenergetic (VMI) images at 40–200 keV and virtual non-contrast images (VNC) are shown. Results for each patient (1–20) individually as well as the ratio between the highest CNR (CNR_max_) and the CNR in the CI (CNR_conv_) are presented. Highest CNR regarding the visibility of the lesions are highlighted in bold. For patient #3, #13 and #16, no value is highlighted as the CNR_max_/CNR_conv_ was lower than 1.5

**Table 4:** Results of the objective image analysis comparing the contrast-to-noise ratio of the lesions’ periphery and normal liver parenchyma in the biopsy images

CNR: periphery of lesion vs. liver	CI	VMI 50	VMI 40	VMI 60	VMI 70	VMI 80	VMI 90	VMI 100	VMI 110	VMI 120
pat #										
1	0.03	**0.78**	0.32	0.02	0.16	0.29	0.37	0.43	0.47	0.49
2	0.20	**2.16**	1.11	0.32	0.07	0.35	0.54	0.70	0.75	0.82
3	0.07	**4.53**	2.35	0.94	0.06	0.53	0.88	1.13	1.31	1.44
4	1.70	**2.91**	2.47	2.18	2.00	1.87	1.80	1.76	1.71	1.68
5	0.89	**6.76**	4.84	.27	2.20	1.48	0.97	0.63	0.37	0.19
6	1.18	0.15	0.79	1.27	1.55	1.74	1.86	1.93	2.01	2.07
7	2.30	**4.43**	3.61	3.10	2.74	2.53	2.39	2.26	2.20	2.18
8	2.46	**4.04**	3.45	3.02	2.81	2.70	2.57	2.51	2.41	2.41
9	0.26	0.03	0.28	0.45	0.54	0.61	0.66	0.67	0.70	0.72
10	1.78	**5.25**	3.80	2.80	2.16	1.74	1.45	1.28	1.14	1.04
11	0.89	1.07	1.31	1.49	1.57	1.61	1.64	1.67	1.68	1.69
12	3.46	4.68	4.44	4.19	4.06	3.94	3.93	3.82	3.84	3.78
13	3.16	4.46	3.94	3.56	3.32	3.16	3.07	2.98	2.91	2.88
14	0.48	**1.22**	0.63	0.22	0.03	0.19	0.29	0.36	0.42	0.45
15	0.11	0.11	0.01	0.10	0.14	0.18	0.19	0.21	0.22	0.22
16	0.16	**1.28**	0.65	0.22	0.06	0.24	0.36	0.44	0.49	0.53
17	0.60	**1.80**	1.31	0.98	0.78	0.64	0.56	0.49	0.46	0.43
18	0.12	**3.93**	2.05	0.81	0.04	0.47	0.80	1.02	1.19	1.31
19	0.09	0.11	0.11	0.11	0.11	0.11	0.11	0.11	0.11	0.11
20	0.57	0.99	0.24	0.22	0.49	0.69	0.78	0.88	0.93	0.96

CNR: periphery of lesion vs. liver	VMI 130	VMI 140	VMI 150	VMI 160	VMI 170	VMI 180	VMI 190	VMI 200	VNC	CNRmax/CNRconv
pat #										
1	0.51	0.53	0.55	0.55	0.57	0.57	0.58	0.59	0.62	26
2	0.93	0.96	1.04	1.00	1.04	1.04	1.04	1.04	1.23	10.8
3	1.56	1.62	1.71	1.70	1.74	1.82	1.82	1.82	2.06	64.7
4	1.66	1.66	1.63	1.65	1.62	1.60	1.61	1.61	1.56	1.7
5	0.03	0.05	0.11	0.18	0.25	0.29	0.36	0.39	0.66	7.6
6	2.12	2.12	2.14	2.18	2.16	2.17	2.23	2.21	**2.27**	1.9
7	2.08	2.05	2.13	2.03	2.07	2.01	2.04	1.98	1.91	1.9
8	2.36	2.39	2.39	2.29	2.33	2.30	2.29	2.29	2.24	1.6
9	0.74	0.74	0.73	0.76	0.76	0.73	0.77	**0.78**	0.76	3
10	0.97	0.93	0.87	0.85	0.79	0.77	0.77	0.77	0.68	2.9
11	1.68	1.69	1.70	1.69	1.70	1.69	**1.71**	1.69	**1.71**	1.9
12	3.76	3.75	3.79	3.72	3.77	3.79	3.75	3.74	3.71	1.4
13	2.88	2.76	2.86	2.73	2.79	2.83	2.74	2.75	2.72	1.4
14	0.49	0.50	0.52	0.53	0.54	0.55	0.56	0.56	0.63	2.5
15	0.23	0.24	0.24	0.24	0.25	0.24	0.24	0.25	**0.26**	2.4
16	0.58	0.59	0.61	0.63	0.64	0.65	0.65	0.65	0.74	8
17	0.40	0.38	0.36	0.36	0.34	0.34	0.33	0.32	0.28	3
18	1.39	1.47	1.51	1.56	1.59	1.61	1.63	1.66	1.85	32.8
19	0.11	0.11	0.11	0.11	0.11	0.12	0.10	0.11	0.11	1.3
20	1.00	1.02	1.05	1.05	1.07	1.06	1.08	1.10	**1.16**	2

CNR for conventional images (CIs), virtual monoenergetic (VMI) images at 40–200 keV and virtual non-contrast images (VNC) are shown. Results for each patient (1–20) individually as well as the ratio between the highest CNR (CNR_max_) and the CNR in the CI (CNR_conv_) is presented. Highest CNR regarding the visibility of the lesions is highlighted in bold. For patient #12, #13 and #19 no value is highlighted as the CNR_max_/CNR_conv_ ratio was lower than 1.5

## Discussion

In this study, the value of virtual monoenergetic and virtual non-contrast imaging derived from spectral data of a DL-CT was evaluated for the visibility of liver lesions during CT-guided biopsy.

In clinical routine, the decreasing visibility of liver lesions over time after application of contrast medium can make CT-guides biopsies more difficult. Even though one can use anatomical landmarks for the navigation during biopsy, false-negative results or repeated biopsies are possible. It seems likely that an increased visibility of the lesion during biopsy makes the procedure easier, accelerates the process, prevents complications and increases the success rate.

In the reader study, an increased visibility via spectral imaging was reported in 16/20 cases. Hereby, VMI-40 (increasing the signal of iodine) was the preferred reconstruction and conventional images were rated worse in all cases. VNC (subtracting the iodine signal) generated the second highest visibility. In four cases, none of the spectral reconstructions was clearly superior to the others or to the conventional images. In accordance with the reader study, the ratio of the CNR between the hepatic lesion and healthy liver could be increased using VMI-40 images in most cases. However, a general recommendation for one reconstruction type cannot be given as there are cases favoring VMI-40 and others in favor of VNC. However, spectral data generate increased visibility in the majority of cases, potentially facilitating CT-guided biopsies.

As either VMI-40 or VNC was the preferred reconstruction in most cases, those reconstructions could be preferably used in the setting of a biopsy. There are different options for the selection of the best reconstruction in the biopsy protocol: first, one could place a ROI in the lesion and in normal liver parenchyma. During the further biopsy, the VMI with the highest CNR should then be automatically selected by the software and the respective image shown during the intervention. Another possibility is to show all VMI levels in the beginning of the biopsy followed by manual selection of the best reconstruction. The latter possibility seems more reasonable until automatic selection is evaluated in a larger study to prevent incorrect selection by the automatic system.

The current study has some limitations, which must be addressed. A relatively small number of patients were included, as the aim of the current study was to evaluate the general feasibility of spectral data in images derived from biopsies. Additionally, only a small number of patients received triphasic CT before biopsy or for staging, which made patient inclusion difficult. Given the promising results of the current study, additional studies with a greater number of patients will be performed. Images were analyzed retrospectively, thus the effect of VMI on the current biopsy procedures could not be evaluated. Due to patient safety, we did not perform a prospective study before proving the potential benefit of the system in a retrospective analysis. Here, further prospective studies with larger patient numbers are needed to evaluate the influence on the outcome of biopsies. Finally, we only used a DL-CT system. We believe that the results of the current study are transferable to other dual-energy systems, but this should be evaluated before application in clinical routine.

## Conclusion

In conclusion, the current study shows a benefit for dual-energy derived virtual monoenergetic and virtual non-contrast images in the setting of CT-guided biopsies. A clear advantage of spectral data for the subjective visibility and objective contrast of liver lesions during biopsy could be shown in the majority of cases. Thus, this system must be further evaluated and then eventually implemented in clinical routine.

## Abbreviations

CI: Conventional images
CNR: Contrast-to-noise ratio
CT: Computed tomography
DE-CT: Dual-energy CT
DL-CT: Dual-layer CT
HU: Hounsfield Units
MRI: Magnetic resonance imaging
ROI: Region of interest
SD: Standard deviation
VNC: Virtual non-contrast images
VMI: Virtual monoenergetic images
